# The Most Effective Molnupiravir Dosage for Eliminating Feline Calicivirus from Cats With and Without Chronic Gingivostomatitis

**DOI:** 10.3390/ani16203265

**Published:** 2026-10-16

**Authors:** Kristina Macaulay, Craig Tebeau, Sarah Green, Stewart McSporran, Diane D. Addie

**Affiliations:** 1 Independent Researcher, Ayr, UKstewart@amiwill.co.uk (S.M.); 2 The Cat Doctor, Federal Way, WA 98023, USA; 3 Healing Spirit Animal Wellness Center, Eureka, CA 95503, USA; 4 Amiwill Ltd., Ayr, UK; 5 Independent Researcher, 64470 Pau, Pyrénées-Atlantiques, France

**Keywords:** gingivitis, EIDD-2801, EIDD-1931, nitazoxanide, mefloquine, fading kittens, calicivirus vaccines, feline calicivirus (FCV) outbreak, gingival hyperplasia, Maine Coon, British Shorthair

## Abstract

Feline chronic gingivostomatitis (FCGS) and gingival hyperplasia are extremely painful conditions for which there is no universally recognised cause or cure. Feline calicivirus (FCV) was detected by RT-qPCR in two pet Domestic Shorthair cats with FCGS and isolated from mouth swabs of 14 cats following a gingivitis and fading kitten outbreak in a British Shorthair and Maine Coon breeding cattery. Oral inflammation was graded 0 (healthy) to 4 (FCGS). Nine of 16 gingivitis cases presented with FCGS. Mean inflammation grade significantly improved (*p* < 0.001) from 3.1 ± 1.0 to 0.3 ± 0.5 when cats became FCV negative. Eleven of 16 (69%) cats gained weight. Cats became FCV negative following antiviral treatment with molnupiravir (n = 15) and nitazoxanide (n = 1). (Nitazoxanide (31–48 mg/kg q24h for 70 days) failed to stop FCV shedding in three other cats.) Across 38 treatment courses, FCV shedding ceased with once daily oral molnupiravir doses of 22–39 mg/kg in 5/10 (50%); 40–55 mg/kg in 14/16 (88%); and >55 mg/kg in 8/12 (67%). Subclinically infected cats became FCV negative after 14 days molnupiravir, but gingivitis cases required longer (*p* = 0.005). Three cattery cats were reinfected and relapsed. Conclusion: Molnupiravir at a dose of 40–55 mg/kg q24h stopped FCV shedding, enabling recovery of some gingivitis cases.

## Simple Summary

Feline chronic gingivostomatitis is an extremely painful and debilitating condition in which the gums and oral mucosae are inflamed, proliferative, and bleeding. Many causes have been suggested, including the cat flu virus, feline calicivirus (FCV). Since calicivirus infection is widespread, it was unknown whether its presence in chronic gingivostomatitis cases was causal or incidental. Many cures have been attempted and until now removal of all healthy teeth, a highly invasive and painful surgical intervention, has been the most successful. In this paper we provide evidence that nine cats with chronic gingivostomatitis and seven with gingivitis recovered or improved when treated for calicivirus. However, three cats were reinfected and relapsed. FCV shedding ceased following treatment with the antiviral molnupiravir in 15 cats, and nitazoxanide in one cat. In this observational case series, the highest proportion of FCV clearance was observed among cats receiving 40–55 mg/kg given by mouth once daily (or divided into two doses). Separating the patient from other calicivirus-infected cats in the same household to prevent reinfection and relapse was essential. Calicivirus-infected cats without gingivitis required only two weeks of antiviral treatment, but cats with gingivitis required longer. A negative FCV test indicated that antiviral treatment could stop, although painkillers may be required longer.

## 1. Introduction

Feline chronic gingivostomatitis (FCGS) is a relatively common, extremely painful and debilitating inflammatory condition of the oral mucosa for which there is no universally recognised cure [1,2]. A meta-analysis of 17 papers concluded that 10.9% of cats may suffer from FCGS [1]. Clinical signs include bleeding gums; halitosis; ptyalism; intense pain when eating or reluctance to eat leading to weight loss; pawing at the mouth; and reduced grooming and consequently an unkempt coat [2,3,4,5]. Hypersalivation is an indicator that cats are in more pain than cats that do not drool [5].

In FCGS inflammation usually extends to the caudal oral mucosa lateral to the palatoglossal folds (“fauces”) [2,6]. The majority of cats with FCGS also have oesophagitis [7]. FCGS differs from other causes of gingivitis in that the teeth often appear healthy, although alveolar bone loss and root resorption can be detected on radiography [8]. Failure to cure FCGS can result in euthanasia [2,9].

Several hypotheses have been offered as the cause of FCGS [3,10,11,12,13,14,15,16,17], but none are universally accepted and aetiology is usually described as “unknown” [1]. Histopathology of lesion biopsies reveals a lymphoplasmacytic infiltrate [3,10,18,19,20]. The predominance of CD8^+^ over CD4^+^ lymphocytes suggests the induction of an underlying cytotoxic cell-mediated inflammatory immune response, consistent with a chronic viral infection [18,19,20].

Although feline immunodeficiency virus has long been associated with FCGS [17,21,22] and feline leukaemia virus has also been implicated [3], feline calicivirus (FCV), a highly contagious unenveloped positive-strand RNA virus [23],was the only microbe strongly associated with FCGS in various studies [10,24,25,26,27,28,29]. However, FCV antigens were not detected in biopsies from FCGS lesions [3], and FCV strains taken from cats with FCGS do not always cause FCGS in experimental infections of laboratory cats [10,30,31].

Historically, immunosuppressive treatments with megestrol acetate [32], corticosteroids [33], or cyclosporin [34] afforded temporary relief but caused adverse effects such as obesity, diabetes, and secondary infections. Newer treatments, some very drastic, invasive, and expensive, include extensive teeth extraction [35,36,37,38,39,40] (which risks puncture of the eyeball [41]), mesenchymal stem cell therapy [39,42,43], or laser therapy [44,45]. FCGS is the reason for 32% of tooth extractions in cats [40].

Few *in vivo* studies of drug efficacy against FCV have been published, other than with recombinant feline interferon omega [33,46,47,48,49]. One study demonstrated that nitazoxanide (NTZ; 2-[(5-nitro-1,3-thiazol-2-yl) carbamoyl] phenyl acetate), an FDA-approved parasiticide, reduced viral titres of four Chinese strains of FCV in experimental kittens [50].

Molnupiravir (EIDD-2801) and its active metabolite EIDD-1931 are nucleoside analogue antiviral drugs shown to be effective against feline coronavirus (FCoV), the virus that causes feline infectious peritonitis (FIP) [51,52,53]. FCoV, like FCV, is a positive-strand RNA virus; therefore, EIDD would reasonably be expected to also have activity against FCV. Cat guardians have been able to obtain unlicensed molnupiravir and EIDD-1931 online for some years [51], from several online vendors that sell variations of the drug in different products without any formal regulatory or veterinary oversight. Similarly to the early days in FIP treatment [51], cat guardians have been treating their own FCGS cases with mixed results. Recently EIDD-1931 became legally available for off-label use under veterinary prescription from compounding pharmacies in some countries including the UK. However, to date, so far as we are aware, and at least in the English language, no peer-reviewed clinical trials exist in the veterinary literature to give guidance on using these antivirals to treat FCV infection or FCGS. The objectives of this paper are to establish the most effective dose and duration of molnupiravir to stop FCV shedding, and to provide evidence that eliminating FCV is the likely mechanism by which antiviral treatment can resolve FCGS.

## 2. Materials and Methods

### 2.1. Cats

Cats from a large breeding cattery experienced an FCV outbreak following a change in vaccine from Purevax RCP^TM^ (Boehringer Ingelheim, Lyon, France) containing two inactivated strains of FCV (FCV 431 and G1) to Nobivac Tricat Trio^TM^, (Merck Sharp & Dohme UK Limited, London, UK) containing live FCV strain F9. A timeline of events in the cattery is shown in Supplementary Table S1.

Infected cats and kittens developed upper respiratory tract disease (URT, “cat flu”), and four kittens died: FCV was isolated [28,54,55] post-mortem from their lungs. Several cats developed gingivitis, some severely; therefore, a search began for an antiviral that would cure them. Two domestic shorthair cats with FCGS in the USA were also treated.

The cattery comprised 17 pens with outdoor runs, allowing the cats to be housed in groups of two to seven cats. Pregnant queens were brought into the house into individual dedicated nursing queen rooms where they could be isolated with their own litter. During antiviral treatment, cats were housed individually or in groups of no more than three concurrently treated cats. Following treatment, cats were not reintroduced to group housing until their FCV-negative status was confirmed. Two ex-cattery cats (Cats 11 and 12) were treated with molnupiravir in their new homes. Seven cats that had been treated for gingivitis were neutered and re-homed to pet households with the new cat guardian being fully informed of their medical history. Where other cats were present in the new home, we requested they be tested for FCV to avoid reinfecting the recovered cat. Advice was given to avoid live calicivirus vaccines to prevent possible reinfection of the recovered cat by vaccinal virus.

The vaccination status of the two FCGS pet cats was unknown for Cat 15, and Cat 16 had not been vaccinated for three years at time of diagnosis.

### 2.2. FCV Detection

Cotton swabs were used to gently obtain saliva from the oral cavity, put into viral transport medium, then mailed first class to the University of Glasgow Veterinary Diagnostic Services laboratory (University of Glasgow Veterinary Diagnostic Services, Glasgow, G78 4AB, UK) for virus isolation in feline embryo A cell culture as previously described [28,54,55]. FCV RNA was tested for by reverse-transcriptase quantitative PCR (RT-qPCR) at IDEXX Laboratory (IDEXX Veterinary Diagnostic Laboratory, Westbrook, ME, USA) and the Real-time PCR Research and Diagnostics Core Facility at the University of California Davis (The Real-time PCR Research and Diagnostics Core Facility of the University of California, Davis, CA 95616, USA). FCV test results were reported as negative when no virus was isolated in cell culture, or no viral RNA was detected on the 40th PCR cycle.

### 2.3. Antivirals

Molnupiravir (Molsun, Sunrise Remedies, Gujarat, India) (Wedgewood Pharmacy, WA, USA), EIDD-1931 (Bova Specials, London, UK) and NTZ (Nizonide: Lupin Ltd, Mumbai, Maharashtra, India) were obtained from compounding pharmacies. After purchase, independent liquid chromatography mass spectrometry analysis (Petcome, Jiaxing City, Zhejiang Province 314006, China) of 200 mg Molsun molnupiravir pills confirmed that they contained at least 195 mg of EIDD-2801.

Effective antiviral doses and treatment duration were assessed empirically.

A bolus of food was given immediately after dosing with pills to prevent oesophagitis.

### 2.4. Gingival Inflammation Grades and Categories

The degree of oral inflammation and gingivitis was graded based on the systems of Hennet et al., 2011 [33], Druet and Hennet, 2017 [37], Matsumoto et al., 2018 [48]; Kim et al., 2023 [56], and Villegas-Ferré et al., 2024 [57].

Grade 0: Normal gingivae (absence of inflammation, no bleeding). (Figures 1c and 3b,f)

Grade 1: Mild erythematous line along the gum above teeth (as in feline juvenile gingivitis-periodontitis, see Figure 2d), no ulceration, no gingival proliferation, and no bleeding, even on gentle probing. 

Grade 2: Mild inflammation/reddening of gums (Figures 2d and 3e), gingival proliferation absent or only slight, no bleeding on gentle pressure or gentle probing.

Grade 3: Moderate inflammation and swelling/gingival proliferation (gingival hyperplasia; see Figures 2b,f and 3a), possible ulceration, no spontaneous bleeding, but bleeding induced by gentle pressure on lesions.

Grade 4: Severe erythema and swelling/gingival hyperplasia, possible ulceration, bleeding on probing and tendency to spontaneously bleed (Figures 2b and 3c). Drooling; crying out in pain on eating or yawning. Cats with this level of inflammation are usually classed as FCGS cases and faucitis is generally present.

Oral inflammatory disease was loosely categorised based on the definitions by Williams and Aller, 1992 [58] with minor alterations:

1. Feline juvenile gingivitis–periodontitis (Figure 2d)
2. Adult-onset periodontitis
3. Adult-onset gingivitis (AOG)
4. Feline gingival hyperplasia (FGH) (Figures 2b,f and 3a)
5. Feline chronic gingivostomatitis (FCGS) (Figures 1a and 2a,e,f)

A photographic record was kept of the cattery cats’ mouths. Cats were described as “recovered” if their mouths could be graded as zero, and “improved” if Grade 1. “Clinical relapse” denoted a return of oral inflammation in a previously recovered cat, which in this case series always followed reinfection by an in-contact cat. The date of clinical recovery (or improvement) was actually the date the mouth examination observation was recorded, although in some cases healing would have occurred sooner but not been observed.

### 2.5. Statistical Analysis

Clinical response efficacy was assessed in the 16 cats that presented with gingivitis, comprising 14 cats from a single UK breeding cattery and two pet cats in the USA. Re-infection occurred in three cats, one of them twice, giving 20 gingivitis episodes in total, of which 19 were treated with an antiviral.

Statistical analysis was performed using Statskingdom online calculator (https://www.statskingdom.com (accessed on 2 July 2026)). Oral inflammation improvement was recorded on an ordinal five-point scale (Grade 0 to 4). Pre- and post-treatment grades were therefore compared using the Wilcoxon signed-rank test. Weight change following successful antiviral treatment (i.e., the cat tested FCV negative post-treatment) was analysed using a two-tailed paired *t*-test. The null hypotheses were that FCV clearance would not improve gum health and that cat weight would not change.

Time to FCV clearance was analysed using pairwise Mantel–Haenszel log-rank tests comparing clearance times between dose groups. Viral clearance was assessed at the treatment-event level (the first assessable treatment event in 38 cats, namely the two DSH pet cats and 36 of 38 purebred cattery cats; the single EIDD-1931-treated cattery cat was excluded, as was one other cattery cat for the reason described in 3.5). Subsequent treatment events were excluded due to the possibility of residual antiviral drug remaining in the body affecting the number of days to viral clearance.

Time to FCV clearance was counted in whole days from and including the first day of dosing. Where the first FCV negative swab was sent on or before the last day of dosing, the count ran to the date that swab was sent; otherwise, it ran to the last day of dosing. Cats that had not cleared were censored on the last day of that course, and no value was substituted. 

Statistical significance was defined as *p* < 0.05.

Figures 4 and 5 were drawn with Matplotlib version 3.10.9 (https://matplotlib.org (accessed on 12 May 2026)) in Python 3.12; data were read from the source workbook with openpyxl version 3.1.5 (https://openpyxl.readthedocs.io (accessed on 12 May 2026).

## 3. Results

Cats 1 to 16 were the gingivitis cases (Cats 1 to 14 from a UK breeding cattery and Cats 15 and 16 were pet cats from the USA). Higher cat reference numbers denote FCV-infected cattery cats that did not develop gingivitis.

When first presented for gingivitis, all cattery cats’ vaccines were up-to-date (Supplementary Table S2).

### 3.1. Resolution of Feline Gingivitis Clinical Signs Following Antiviral Treatment

Sixteen cats had gingivitis; nine severe enough to be classified as FCGS. Thirteen cats clinically recovered (Figure 1, Figure 2 and Figure 3, Table 1) and three improved after eliminating FCV using molnupiravir in 15 cats and NTZ in one cat (Figure 2). However, cats 5, 6, and 8 were reinfected and relapsed (while still living in the cattery), giving a total of 20 gingivitis episodes, 19 of which were successfully treated with antivirals (Table 1).

**Figure 1:**
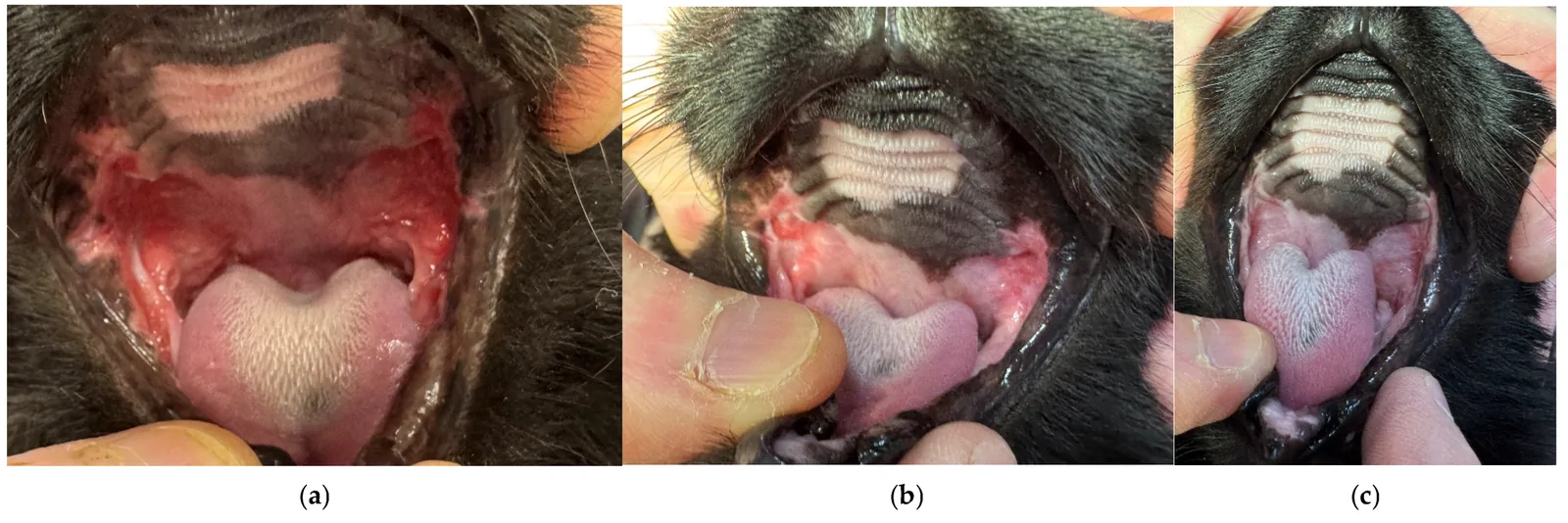
This figure shows Cat 16’s chronic gingivostomatitis recovery with molnupiravir. Cat 16’s history: despite full mouth extraction 4.2 years previously, tissue ablation surgery, various antibiotics, dexamethasone, high-dose (14 mg/kg q24h) cyclosporin (Atopica Elanco US Inc., Indianapolis, IN USA) and daily buprenorphine for analgesia, his oral comfort and appetite remained poor. His weight had continued to decline from 5.62 kg at time of the extractions to 4.66 kg on the day molnupiravir treatment began. (**a**) Weight 4.66 kg. Grade 4 inflammation. Photographed just prior to starting molnupiravir therapy showing the intense erythema, friability, and ulceration of the caudal oral mucosa (“fauces”) characteristic of FCGS. Compounded liquid molnupiravir (20 mg/kg q12h) was prescribed to be administered orally. At veterinary examination on Day 16 of molnupiravir (no photograph) his caregiver reported notable clinical improvement; he was showing fewer signs of oral pain (e.g., he could yawn without crying out), his appetite had improved and he was showing increased interest in food. His weight had risen to 4.72 kg, an increase of 60 g; this improvement represented a significant reversal of the progressive weight loss that had characterised the previous year. (**b**) 35 days after starting molnupiravir. Swab taken for FCV test: RT-qPCR negative. Weight 4.99 kg. Grade 1 inflammation. His guardian reported that he was eating more readily, and behaving more normally at home. This visit marked a pivotal turning point in the cat’s management, demonstrating a robust and clinically meaningful response to antiviral therapy after years of refractory disease. Buprenorphine and cyproheptadine were discontinued, and cyclosporin dose reduced to 7 mg/kg per day. (**c**) Cat 16, 48 days after starting molnupiravir. Weight 4.99 kg. Grade 0. Results of full bloodwork, urinalysis, and abdominal ultrasound were all within normal limits. All medications were discontinued. Eighteen months after completing antiviral treatment (no photograph) the cat’s mouth remains comfortable and his quality of life excellent. His weight remains stable at 4.99 kg.

**Figure 2:**
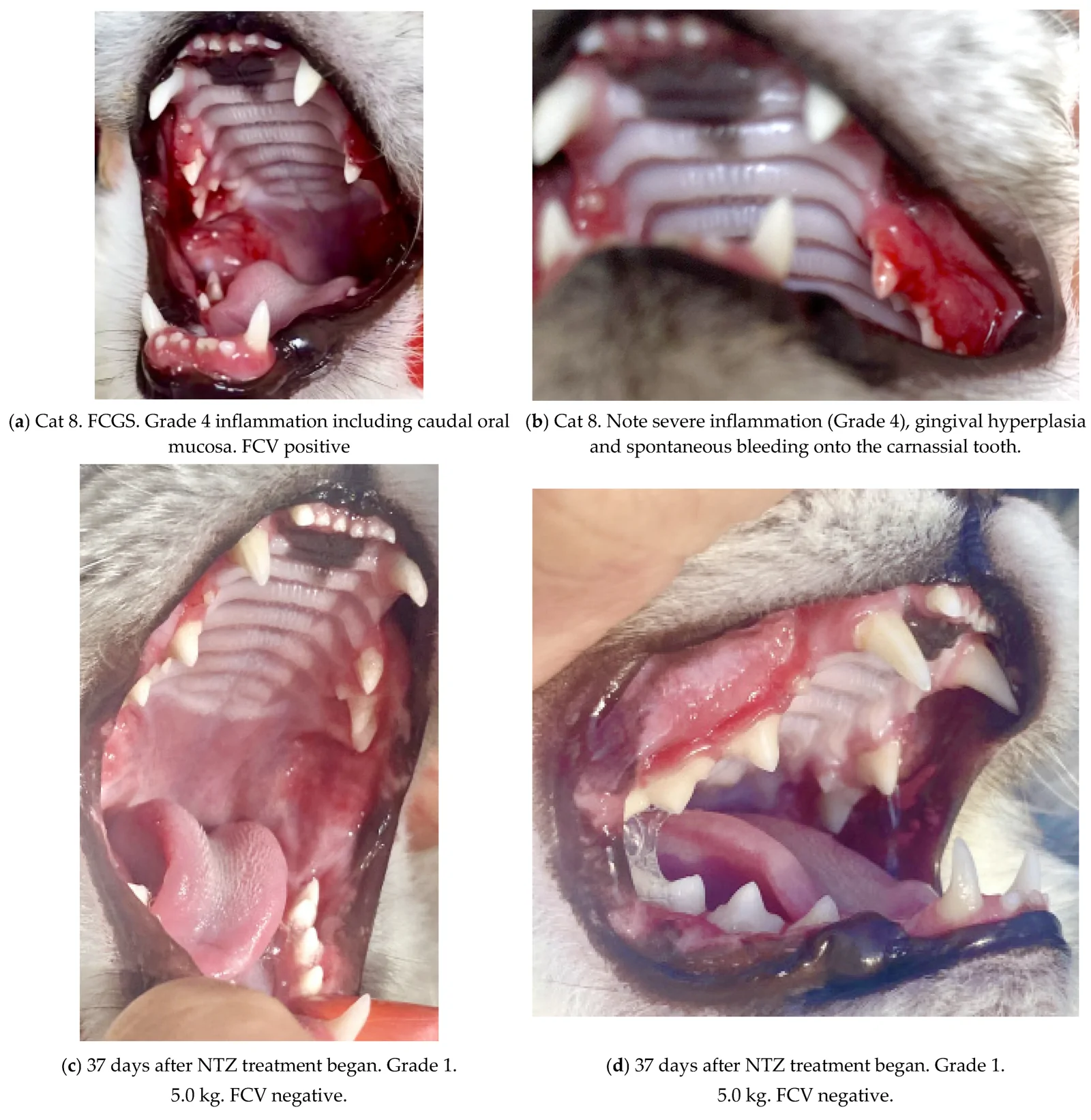

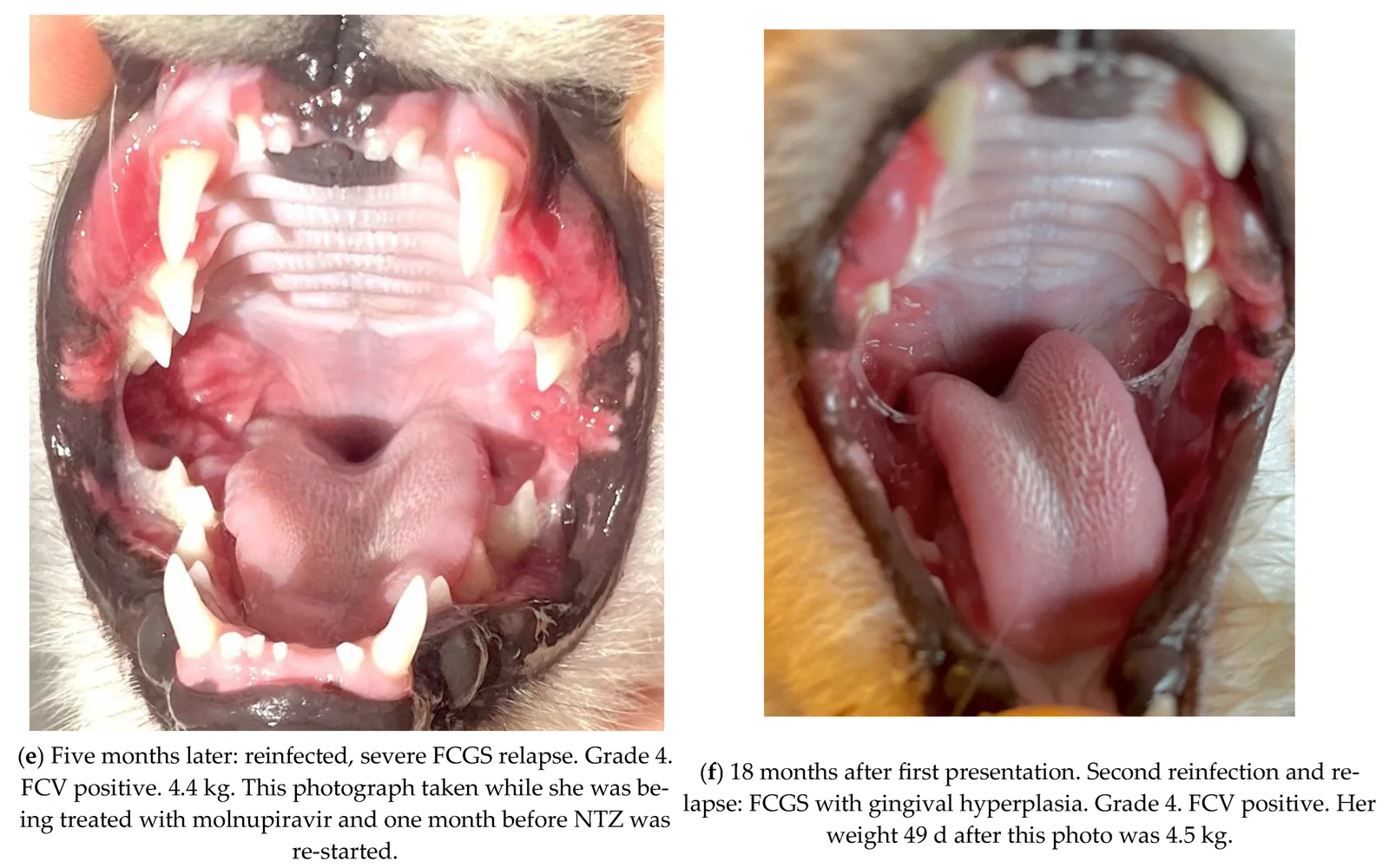
Cat 8, a female Maine Coon, presented with severe feline chronic gingivostomatitis (FCGS) at 9 months of age. These photographs document Cat 8’s two recoveries then two relapses after calicivirus reinfections. Photographs (**a**,**b**) were taken 45 d before NTZ treatment began: she was FCV positive. Although not weighed on day of the photographs, she weighed 3.9 kg when NTZ began. (**a**) Inflammation Grade 4. (**b**) Close up of left upper carnassial showing severe gingival hyperplasia and spontaneous bleeding of the gums. (**c**,**d**): Considerable improvement following nitazoxanide (NTZ) treatment (32 mg/kg q24h for 37 d). These photographs show reduction in inflammation and resolution of gingival hyperplasia. No calicivirus was isolated from a swab taken on the day of this photograph. She had gained 1.1 kg. (**e**) Following reinfection, the cat relapsed with severe (Grade 4) gingivostomatitis. This photo clearly shows inflammation of caudal oral mucosae, gingival proliferation, and loss of incisors. Treatment began with low-dose molnupiravir (31 mg/kg q24h for 31 d). The daily dose of molnupiravir was increased to 44 mg/kg, then a high dose (65 mg/kg) (a high dose was defined as a dose over 55 mg/kg). One month after photograph (**e**) the mouth appeared recovered grossly but radiography revealed bony changes around the incisors and canines, and a neck lesion was evident on the right lower carnassial tooth. Osteomyelitis was suspected, therefore a 10-day course of clindamycin was prescribed, and a second NTZ course (28 mg/kg q24h) was added to molnupiravir for 20 days, after which she tested FCV negative. (**f**) The cat was reinfected and relapsed again; NTZ was not used again due to adverse effects of vomiting and diarrhoea. However, gingivitis persisted, and doxycycline was administered for 28 days. Treatment with depo-corticosteroids was instigated; calicivirus was isolated repeatedly. She remains FCV positive and under treatment at time of writing.

**Figure 3:**
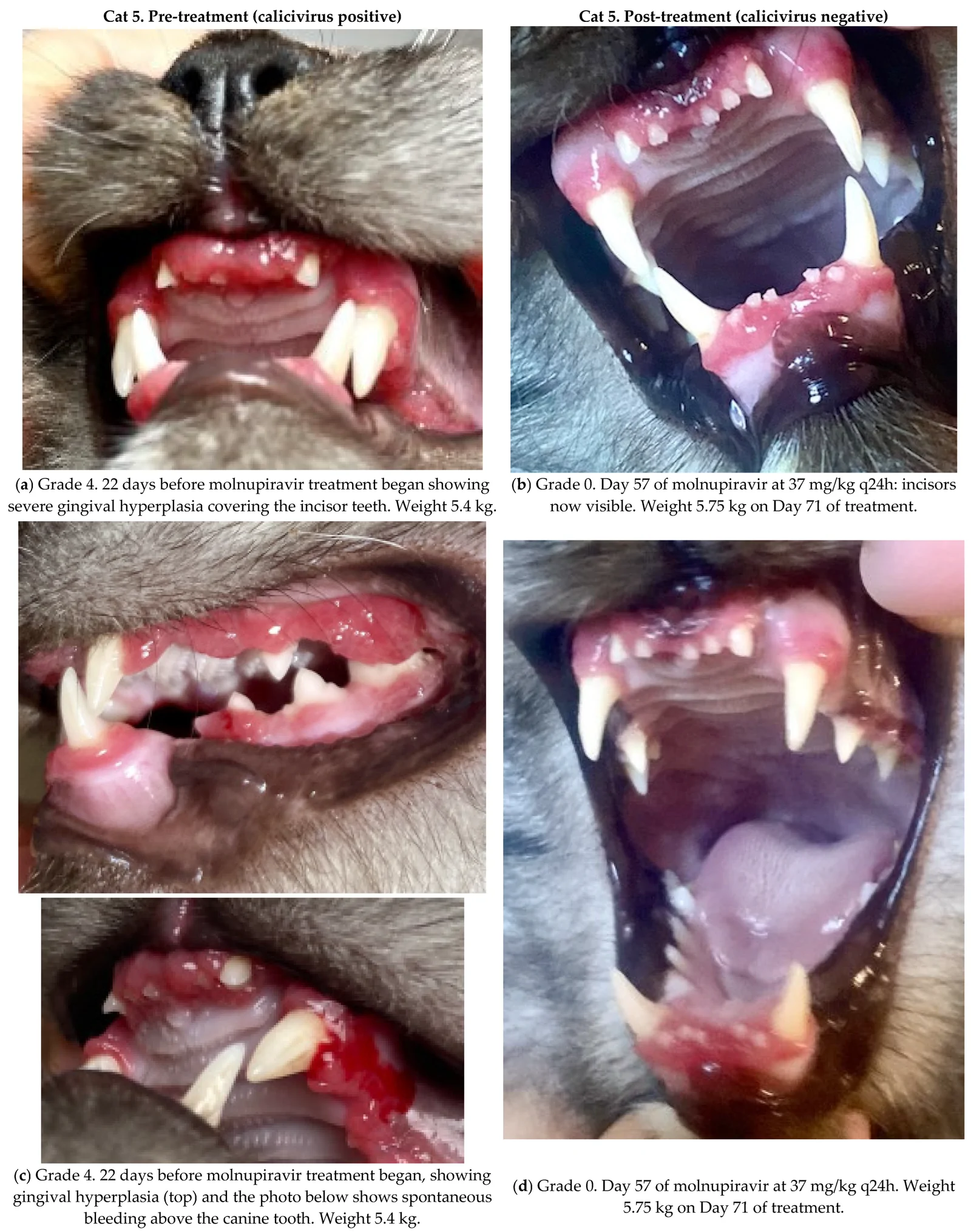

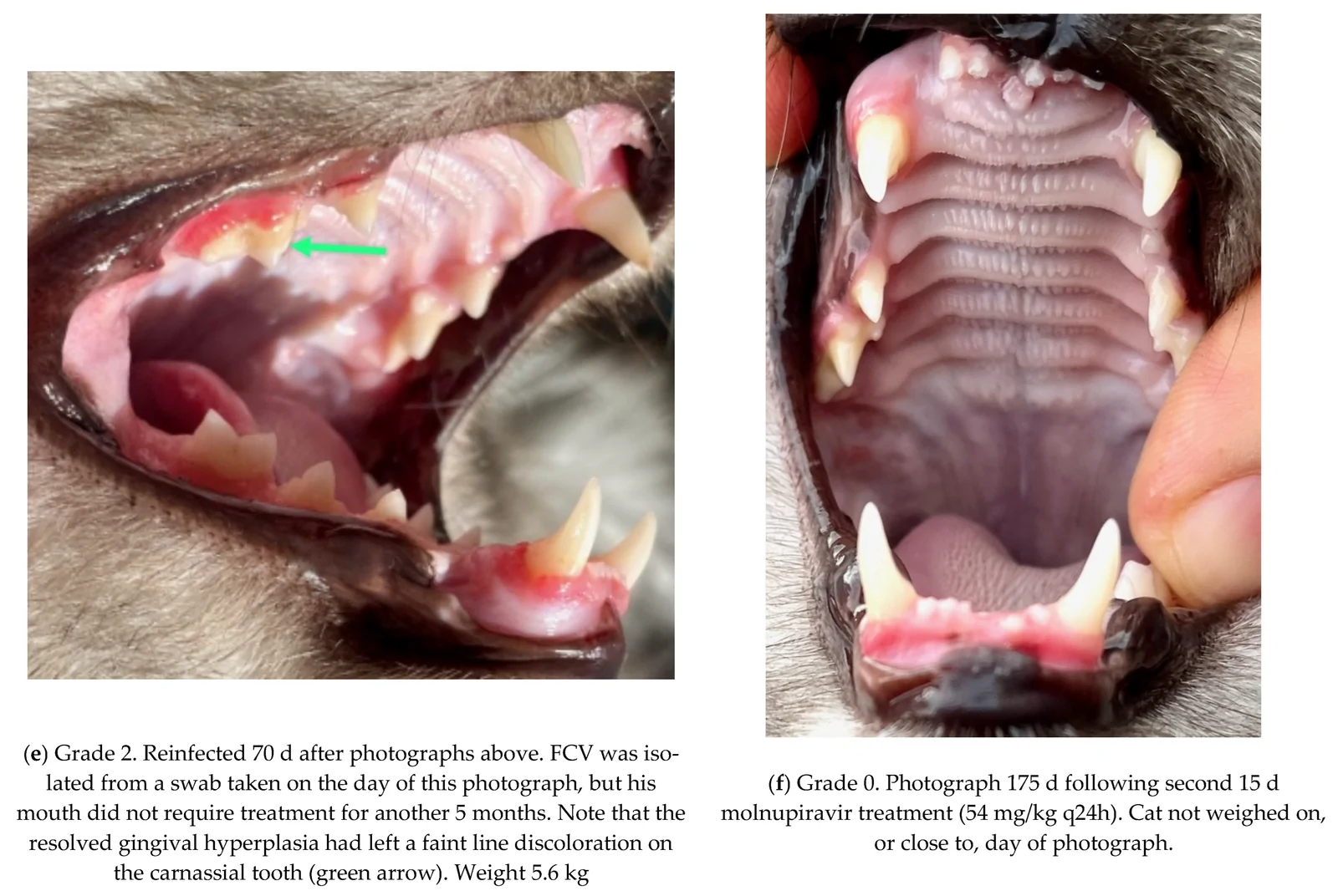
This figure illustrates the case history of Cat 5, a male Maine Coon who presented with feline chronic gingivostomatitis (FCGS) and gingival hyperplasia (FGH) at 7 months of age. Feline calicivirus (FCV) was isolated. The column on the left shows his mouth before, and on the right after, molnupiravir treatment. (**a**) Gingival hyperplasia showing that only three upper incisors were visible prior to molnupiravir treatment. (**b**) After 57 days of molnupiravir (37 mg/kg q24h) the five remaining incisors were visible. (**c**) The same cat’s pre-treatment lateral mouth showing severe gingival hyperplasia (top photo) and spontaneous bleeding to right of the upper canine tooth (lower photo). (**d**) Mouth healed and gingival hyperplasia resolved. (**e**) FCV testing showed that he was reinfected within 70 d; yet his mouth relapsed five months after becoming reinfected, and with less severe inflammation than previously, but he had lost 150 grams of weight. He was re-treated with molnupiravir (54 mg/kg q24h for 15 days). (**f**) This photograph shows the cat’s mouth still completely healthy at six months after his second molnupiravir treatment.

**Table 1:** Gingivitis severity before and after molnupiravir (EIDD-2801) treatment of 16 cats.

Cat Reference and Breed	Age (Years) When Gingivitis Diagnosed	Duration Gingivitis Prior to Molnupiravir	Treatment History Before Molnupiravir	Dose of Molnupiravir That Eliminated FCV	Inflammation Grade	Faucitis	Gingival Hyperplasia	Bleeding	Tooth Loss ^a^	Weight Change (g)	Gingivitis Class and Outcome Post-Treatment
1 MCO	1.0	1.6 y	Dental examination found teeth were healthy: no extractions. Biopsy: lymphoplasmacytic gingivitis. Mefloquine. Amoxycillin clavulanate. Clindamycin. Meloxicam. Azithromycin.		4	x	✓	✓	x 6/6		**FCGS**
1				33 mg/kg q24h, 29 d	0	x	Resolved slowly.	x	✓6/2	0	Recovered and still FCV negative 7 w later.
2 MCO	1.2	6 m	NTZ (36 mg/kg q24h for 70 d). Azithromycin.		4	✓	✓	✓	✓4/4		**FCGS** FCV positive on 4 consecutive tests over 198 d.
2				57 mg/kg q24h, 20 d	0	x	x	x	x 4/4	550	Recovered: still FCV negative 1 m later and well 9 m later.
3 ^b^ BLH	1.0	2.5 m	NTZ (48 mg/kg q24h for 70 d: see note ^b^). Azithromycin.		3	x	✓	x	x 6/6		**FGH** FCV positive on 2 consecutive tests over 71 d.
3				77 mg/kg q24h, 10 d	0	x	x	x	x 6/6	150	Recovered: 5 w follow-up.
4 MCO	4.5	1 d	Azithromycin.		4	U	✓	x	✓5/5		**FCGS**
4				56 mg/kg q24h, 60 d	0	x	x	x	✓4/3	400	Recovered very slowly (G1 inflammation on Day 287, G0 on Day 392). Remained FCV negative.
5 MCO	0.6	5 m	Clindamycin. Azithromycin.		4	x	✓	✓	✓5/6		**FCGS:** see Figure 3.
5				37 mg/kg q24h, 72 d	0	x	x	x	✓5/5	350	Recovered post molnupiravir, but reinfected 2 m later and showed FGH 5 m after reinfection.
5-r	1.6	0 d ^d^	No: molnupiravir began when diagnosed		2	x	✓	x	x 5/5		**FGH.** Grade 2 retreated.
5-r				54 mg/kg q24h, 15 d	0	x	x	x	✓ 5/4	NA	Recovered and still well 6 m later.
6 BSH	2.4	2.5 m	NTZ (31 mg/kg q24h for 70 d: still FCV positive). Azithromycin.		4	✓	✓	✓	x 6/6		**FCGS** FCV positive on 4 consecutive tests over 102 d.
6				50 mg/kg q24h, 25 d	1	x	Mostly resolved	x	x 6/6	−150	Recovered, but reinfected and relapsed after 195 d. Weight loss explained by lactating at the time.
6-r	3.3	17 d	No; molnupiravir began after positive FCV test reported.		4	✓	✓	✓	✓5/2		**AOG**
6-r				41 mg/kg q24h, 15 d	1	x	x	x	✓ 5/2	0	Improved but not fully cured (Grade 1) when rehomed 3 m later.
7 BSH	2.4	5 d	Radiography and GA dental examination with extractions 11 d before molnupiravir. ^e^ NTZ (40 mg/kg) for 7 d (discontinued because she vomited it).		2	✓	✓	x	0/0 Extracted ^e^		**FCGS**
7				32 mg/kg q24h, 66 d	0	x	Resolving	x	NA	850	Recovered. Still FCV negative and mouth healed when tested 5 w later. Cat then sold.
8 ^c^ MCO	0.8	>5 m	Clindamycin and prednisolone (at least 33 d).		4	✓	✓	✓	x 6/6		**FCGS:** see Figure 2.
**8**			FCV negative after 37 d of NTZ ^c^ (32 mg/kg).	Treated with NTZ, not molnupiravir	1	x	x	x	✓6/5	1100	Recovered and FCV negative post NTZ, not molnupiravir, but reinfected within 3 m: severe relapse.
8-r	1.1	36 d	Treated with molnupiravir for 165 d. GA dental, full mouth radiography. Clindamycin 8 mg/kg q12h 10 d.		4	✓	✓	✓	x6/5		**FCGS relapse:** Still FCV positive on 6 of 6 tests over 129 days of a 165 d molnupiravir course until NTZ was added to treatment.
8-r1			NTZ (28 mg/kg q24h) added for the last 20 d of the molnupiravir course before the FCV negative result.	NTZ ^c^	0	x	x	x	✓6/4	600	Recovered briefly grossly (abnormalities revealed on radiography). However, was reinfected within weeks, and severely relapsed again.
8-r2			Not re-treated with NTZ due to vomiting and diarrhoea adverse effects: being treated with antibiotics and corticosteroids.		4	✓	✓	✓	✓4/4		**Second reinfection and FCGS relapse** Not cured: still FCV positive and with FCGS 8 m later.
											
9 BSH	3.2	0 d ^d^	No: molnupiravir began when diagnosed.		2	x	✓	x	✓5/6		**FGH**
9				59 mg/kg q24h, 15 d then 10 d	0	x	x	x	✓4/6	0	Recovered: 4 m follow-up.
10 BSH	3.1	0 d ^d^	No: molnupiravir began when diagnosed.		2	x	✓	x	x 6/6		**FGH**
10				67 mg/kg q24h, 16 d then 60 mg/kg q24h, 10 d	0	x	x	x	✓6/5	320	Recovered: 23 d follow-up.
11 BSH	3.6	3 m	FGH diagnosed on day presented for booster vaccine. Metronidazole. Meloxicam.		2	x	✓	x	✓ 5/6		**FGH**
11				54 mg/kg q24h, 6 d	1	x	x	x	✓ 5/6	500	Improved to G1 at 22 d, reported recovered at 2 m. 9 m follow-up.
12 MCO X	1.9	15 d	No: molnupiravir began when diagnosed.		3	x	✓	x	✓ 6/5		**FGH**
12				71 mg/kg q24h, 20 d	1	x	x	x	✓ 6/5	1500	Improved: mild inflammation remained 10 w later and 3 more incisors lost (4/4).
13 BSH	1.1	0 d ^d^	No: molnupiravir began when diagnosed.		1	x	✓	x	x 6/6		**FGH**
13				39 mg/kg q24h, for 16 d; 19-day gap, then 40 mg/kg q24h, 5 d	0	x	x	x	x 6/6	0	Recovered: fully healed at 3 m follow-up.
14 MCO	0.8	0 d ^d^	No: molnupiravir began when diagnosed.		2	x	✓	x	x 6/6		**FGH**
14				77 mg/kg q24h, 14 d then 51 mg/kg q24h, 14 d	0	x	x	x	✓6/3	0	Recovery slow post-treatment; G0 d396. No weight gain immediately post-treatment, 9 m later she had gained 500 g. Mouth normal 13 m post-treatment.
15 DSH	1.9	10.5 m	Mandibular molars and premolars extracted 2 m prior to molnupiravir. Buprenorphine; corticosteroids; phototherapy; doxycycline.		4	✓ A little	✓	✓	✓0/1		**FCGS** FCV RNA C*_T_* 37.2 (a very low viral load).
15				40 mg/kg q24h, 30 d	1	x	x	x	✓0/1	491	Much improved: reported well 8 m later. At 17 months post-treatment GA radiography, one extraction, no stomatitis.
16 DSH	4.6	>9.6 y	FME 4.2 y prior. Dexamethasone (adverse event became diabetic); cyclosporin; hydrolysed diet; azithromycin; doxycycline; buprenorphine; tissue ablation surgery of his fauces.		4	✓	U	U	FME		**FCGS**
16				20 mg/kg q12h, 48 d (but FCV negative on Day 35)	0	x	x	x	NA	330	Recovered: still well 18 m later.
**Totals 16 cats**						**9/19 (47%)**	**19/19** **(100%)**	**9/19** **(47%)**	**13/15 (87%)** **cats lost teeth**	**11/16 (69%) cats gained weight**	**20 gingivitis episodes** **(11 FCGS)**

✓ Present. x Absent. AOG Adult-onset gingivitis. BLH British longhair. BSH British shorthair. C*_T_* Cycle threshold. d Days. m Months. DSH Domestic shorthair. MCO (X) Maine Coon (cross). FCGS Feline chronic gingivostomatitis. FCV Feline calicivirus. FGH Feline gingival hyperplasia. FME the cat had already undergone a near full-mouth extraction (leaving canine teeth). GA General anaesthetic. NA Not applicable. NTZ Nitazoxanide. U Unknown. a. Number incisors remaining upper jaw/Number incisors remaining bottom jaw. b. Cat 3 was still FCV positive after 59 days of NTZ, but did not test negative after Day 72, until she had been on 10 days of high-dose (77 mg/kg) molnupiravir. Therefore, we could not rule out that her negative FCV result was due to NTZ rather than molnupiravir, however unlikely. c. See Figure 2. Cat 8 became FCV negative following NTZ treatment. She was reinfected after being moved to a pen with another cat and kittens; her gums began to be inflamed again flaring once more into full-blown FCGS (Figure 2). She was reinfected and relapsed a second time and remains symptomatic at time of writing. d. Treatment (antiviral and such other treatments as deemed necessary by the attending veterinary surgeon) began same day gingivitis was first noticed. e. Radiography and dental 11 days prior to attempted NTZ treatment (cat vomited NTZ). Loose incisors and carnassial teeth were removed: 3 mm and 5 mm gingival pockets.

Table 1 summarises the key information of 16 cats with gingivitis treated with molnupiravir. The first column gives the cat’s reference number and breed; the second shows the cat’s age at the time when gingivitis was diagnosed and the third shows the duration that the feline calicivirus-infected cats had suffered gingivitis. The previous treatments (if any) that had failed to cure the cats are shown in the fourth column. NTZ only stopped FCV shedding in Cat 8; three other cats treated with NTZ remained infected. The nitazoxanide dose is shown in parenthesis. Some cats had suffered for a considerable time and undergone painful and invasive procedures in attempts to cure them. The cat’s clinical condition pre-molnupiravir is shown in the shaded row and after treatment in the clear row underneath: all except Cat 8 tested FCV negative post treatment. The first 14 cats were tested by virus isolation, and the last two by RT-qPCR. Once it was clear that molnupiravir could cure FCV-positive gingivitis, molnupiravir was administered as soon as a diagnosis was made. The fifth column gives the antiviral dose (molnupiravir except for Cat 8) calculated by dividing the antiviral dose administered by the cat’s weight at the start of each course shown, and treatment duration that stopped FCV shedding. The sixth column shows the gingivitis inflammation grade (as defined in Section 2.4) while the cat was FCV positive (row shaded grey) and negative (clear row). The presence or absence of specific clinical signs of gingivitis are then given. The last row shows the total of each clinical sign over the total number of episodes recorded for that sign. The tenth column shows the number of incisors present before treatment began, i.e., whether there had been tooth loss due to gingivitis (shaded row), and the number of incisors remaining after FCV treatment (clear row). The eleventh column shows the number of grams gained or lost after antiviral treatment. The last column indicates the gingivitis classification pre-treatment in the grey row and below, in the unshaded cell, the outcome for each case and duration of follow-up. Nine cats had FCGS, and seven FGH. Three cats were reinfected and relapsed (indicated by -r after their reference number). Cat 8 (Figure 2) was the only gingivitis cat in which molnupiravir did not clear FCV; she was cured of both the virus and clinical signs with NTZ but she was reinfected twice and relapsed.

Across 19 treated gingivitis episodes, median inflammation grade reduced from 4 (IQR 2–4; range 1–4) before treatment eventually to 0 (IQR 0–1; range 0–1) afterwards (Hodges–Lehmann 95% CI: 2.0–3.5). Results of the Wilcoxon signed-rank test indicated that the mean oral mucosal inflammation grade improved significantly (*p* <0.001; *r* = 1.00) from 3.1 (SD ± 1.0) to 0.3 (SD ±0.5) after 19 antiviral treatments.

Molnupiravir was more effective in both reducing oral inflammation and eliminating FCV than nitazoxanide at the doses used (15/16 vs. 1/4 cats). The four cats given nitazoxanide were subsequently given molnupiravir, therefore, the two groups are not independent. 

Excluding relapse episodes and gingivitis episodes for which antiviral treatment was started within one day of gingivitis diagnosis, pre-treatment durations of suffering were available for 11 gingivitis episodes; the median was 150 days (IQR: 75–250). The median for those same cats between the start of antiviral treatment and being recorded as recovered (inflammation Grade 0) or improved (Grade 1) was 37 days (IQR 21–54).

Cats 5 and 6 were reinfected two and six-and-a-half months after their previous recovery, but Cat 5’s relapse occurred five months after his reinfection. When gingival inflammation was noticed, a 15-day molnupiravir course was initiated, and the cats improved. Cat 6 was subsequently re-homed and her mouth has remained healthy since. Details of Cat 8’s relapse are explained in Figure 2 and Section 3.6.

None of the six cats (Cats 7, 9, 10, 12, 15, 16) living in single cat households relapsed, nor did Cats 1 and 2, which were re-homed together as the only pets. Cat 11 was re-homed to a home with one other cat who was also treated for FCV to prevent reinfection of Cat 11 and, to our knowledge, has not relapsed. 

### 3.2. A Breakdown of Clinical Signs of Cats with Gingivitis

Clinical signs were not always fully recorded for all 20 gingivitis episodes; nine of 19 (47%) episodes had faucitis (inflammation of the caudal oral mucosae) (Figure 1 and Figure 2); 19/19 (100%) gingival hyperplasia (Figure 2b,f and Figure 3a); 9/19 (47%) bleeding gums (Figure 2b and Figure 3c). Thirteen of 15 (87%) cats had spontaneously lost incisor teeth (Table 1) (Figure 2e and Figure 3b). Cat 16 was excluded from this last count because he had already undergone full dental extractions, but Cat 7 was included because even though the incisors and carnassial teeth had been removed surgically, those teeth were mobile. Cat 1 had a dental examination with no extractions because under general anaesthetic his teeth were found to be healthy, but by the time molnupiravir became available he had lost four incisors.

### 3.3. Weight Gain Following Antiviral Treatment

Paired weight data before and after FCV elimination were available for only 18 of the 19 treated gingivitis episodes of the 16 cats and included the weights of Cats 6 and 8 before and after relapse treatments (Table 1, Figure 4). Cats gained weight after 12 successful antiviral treatment courses, and their weight was unchanged after five courses; Cat 6 lost weight after her first treatment (likely due to lactation) and her weight was unchanged after her second treatment. Results of the paired t-test indicated that there was a significant (*p* = 0.001) average weight gain of 388 ± 431 g, (median 340 g; IQR 0–538 g), when cats with gingivitis eliminated the FCV infection. 

As shown in Table 1 and Figure 4, resolving FCV-associated gingivitis produced a sustained improvement in body weight in eleven cats. This result was consistent with the clinical expectation that resolving oral pain allows cats to eat more comfortably.

### 3.4. Feline Calicivirus Test Results Summary

Forty-six pedigree UK cattery cats and two USA pet cats (Cats 15 and 16) with FCGS were FCV tested. Eight cattery cats were FCV negative. FCV was isolated from 14 cattery cats with gingivitis (two also had URT signs), and 24 non-gingivitis cases (21 subclinically infected cats and three more with URT signs).

Fourteen of 38 (37%; 95% CI 22–54) FCV-infected cats in the breeding cattery developed gingivitis; seven were Maine Coons.

### 3.5. A Daily Molnupiravir Dose of 40–55 mg/kg Stopped Feline Calicivirus Shedding in 88% of Cats 

In the absence of published guidelines for treating gingivitis with molnupiravir, establishing an effective dose to eliminate FCV was done empirically. In practice only an approximation of the desired dose can be achieved using a combination of whole and partial pills because molnupiravir 200 mg pills can usually only be broken into halves or quarters. The exact dose in milligrams per kilogram (mg/kg) administered to each cat was calculated by dividing the antiviral dose given by the cat’s weight at the start of the cat’s first molnupiravir treatment (Table 2). Cats were treated once a day, except Cat 16 who was treated with 20 mg/kg twice a day.

The breeding cattery instituted FCV testing of pregnant and lactating cats to prevent further kitten losses. In addition to 16 cats with gingivitis, 24 cats without gingivitis tested FCV positive and were treated (Table 2, Figure 5). When EIDD-1931, the active metabolite of molnupiravir, became available, Cat 40 was treated with 32 mg/kg *per os* q24h for 11 days, and became FCV negative. The EIDD-1931 treated cat was excluded from statistical analysis. Cat 3 was excluded from statistical analyses because she tested FCV positive 12 days before the end of her 70-day NTZ course, but was not confirmed still positive prior to molnupiravir beginning. Only the first assessable molnupiravir course of the 38 cats was included in statistical analysis.

**Table 2:** Feline calicivirus clearance at different molnupiravir doses.

Dose Group	mg/kg	Number of Treatments	FCV Negative (%)	Median Days	Range Days to FCV Negative	IQR
Low	22–39	10	5 (50%)	16	8–72 *	10–31
Most effective	40–55	16	14 (88%)**	14	6–60 ***	8–24
High	>55	12	8 (67%)	15	7–20	14–16

FCV: feline calicivirus, IQR: inter-quartile range, * The long durations of treatment were in cats suffering from FCGS. ** Cat 38 was only treated for eight days, when the FCV positive test result arrived after a gap of seven days she was treated again for six days and stopped shedding virus. Although both courses were 44 mg/kg only the first course of eight days was counted. *** Cat 4 was not tested before 60 days: virus shedding likely ceased before 60 days. Two more outliers were FCGS cats treated for 30 and 48 days and not tested earlier than 24 and 35 days after treatment began. Cat 19 tested FCV positive, but received only 15 treatments of 42 mg/kg over a 24-day period. Cat 31 received 31 mg/kg for two days before the dose was doubled. He had tested FCV positive on Day 1 of treatment, but was not tested on Day 3, so the two-day period could not be assessed and his first assessable course was the 63 mg/kg course.

Table 2 shows the effect of varying doses of molnupiravir on the median number of days required to stop FCV shedding in 38 cats. Only the first molnupiravir course of each cat was counted, and increased doses or subsequent treatments were excluded. The highest proportion of FCV clearance was observed among cats receiving 40–55 mg/kg, which was administered once per day in all cats, except split into two doses for Cat 16. 

**Figure 5:**
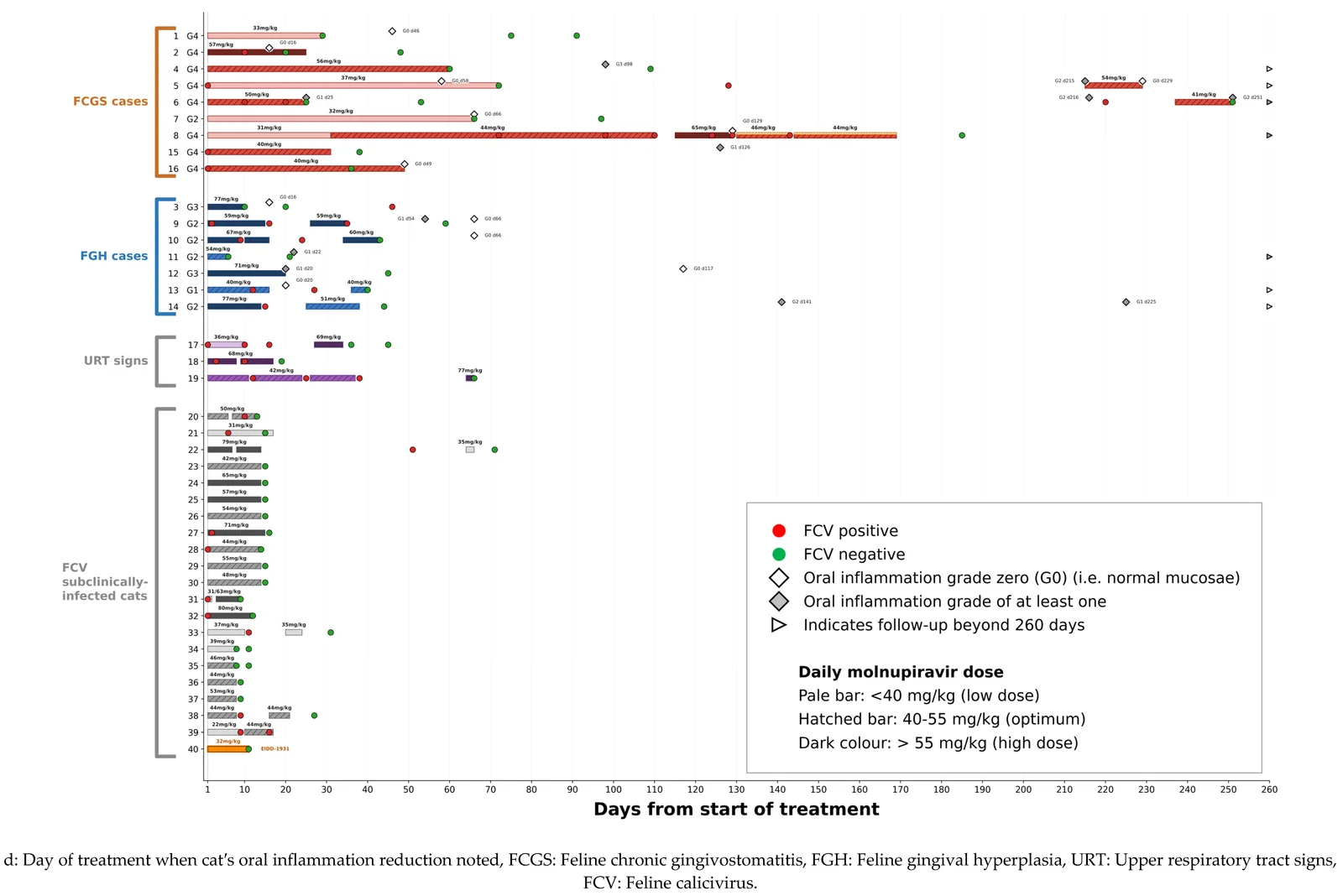
Figure 5 shows the number of days of molnupiravir treatments (represented by bars) given to the 37 cattery cats and two pet cats (Cats 15 and 16). Cattery Cat 40 (the only cat treated with EIDD-1931) is also shown. Cats are grouped into their clinical presentations. The cats’ reference numbers are on the y axis with the pre-treatment grade (G1–G4) of inflammation (see Section 2.4) indicated beside the y axis for the FCGS and FGH groups. The diamond shapes signal at which day of treatment clinical recovery or improvement was first noted. The red and green dots represent positive and negative FCV test results: after a positive test cats were re-treated until they tested negative. Only those positive FCV tests taken on or the day before the first day of treatment are on the y axis. Cats 5, 6 and 8 became reinfected (but Cat 8’s reinfection FCV-positive test was on Day 337, beyond the end of the x axis). Cat 8’s FCV negative test (on Day 185) was not attributed to molnupiravir, but to the second course of nitazoxanide (begun 129 days after she started molnupiravir). The calicivirus strains infecting Cat 8 and possibly Cat 39, which were initially treated with a low dose, may have become resistant to molnupiravir. Cats without gingivitis or URT disease tended to require shorter courses of the antiviral to become FCV negative.

Initially, cats were treated with low doses (under 40 mg/kg q24h) of molnupiravir in case of adverse effects, but low doses cleared the virus from only five of 10 (50%) cats (Table 2, Figure 5). A molnupiravir dose of 40–55 mg/kg q24h was significantly more effective (Log-Rank *p* = 0.015) than doses of under 40 mg/kg to effect virus clearance. High doses (over 55 mg/kg q24h) offered no advantage over a daily dose of 40–55 mg/kg (Log-Rank *p* = 0.95).

### 3.6. Low Dose of Molnupiravir May Have Led to Emergence of Caliciviruses Resistant to Molnupiravir

Ten cats were treated with a molnupiravir dose under 40 mg/kg q24h (Table 2). Five cats became FCV negative; Cats 1, 5, 7, 21, and 34 were treated for 29, 72, 66, 15 and 8 days.

Cats 8, 13, 17, 33, and 39, which continued to shed calicivirus were re-treated, usually with a higher dose of antiviral (Figure 5). The viruses infecting Cat 8, and possibly Cat 39, may have become resistant to molnupiravir.

Cat 8 had previously tested FCV negative and clinically recovered with a 37-day course of NTZ (32 mg/kg q24h) (Figure 2). However, she became reinfected and relapsed and was treated with 31 mg/kg q24h molnupiravir. FCV was isolated from a swab taken on Day 31; therefore, the daily dose was increased to 44 mg/kg, then 65 mg/kg, but she continued to shed virus over a total of six consecutive tests spanning 129 days until NTZ, (28 mg/kg q24h for 20 days) was added to her molnupiravir treatment; at the following test FCV could no longer be isolated. Although molnupiravir had not cleared her calicivirus infection, her oral inflammation appeared grossly to have resolved prior to NTZ beginning, but dental radiography revealed bony changes. Unfortunately, she was reinfected a second time, and relapsed again. Cat 8 was on molnupiravir for a total of 165 days, yet haematology and biochemistry test results were unremarkable except for a raised alpha-1 acid glycoprotein of 3029 µg/mL and an FCoV antibody titre of 1280. She is still being treated at the time of writing.

### 3.7. Cats with Gingivitis Required a Longer Molnupiravir Course than Subclinically Infected Cats

Seventeen of the 21 subclinically infected cats were on a daily dose of at least 40 mg/kg of molnupiravir. Fourteen of the 17 cats became FCV negative on molnupiravir courses of 15 days or fewer, and the three remaining cats required further treatment (Figure 5). 

Excluding cats on low molnupiravir doses of under 40 mg/kg and cats that were not FCV tested before 25 days, six of 10 cats with gingivitis required a molnupiravir course of at least 20 days. Compared with subclinically infected cats, cats with gingivitis required a statistically significantly longer duration of treatment of at least 20 days (Fisher’s exact test, *p* = 0.005; 95% CI: 2.1–244.9). 

## 4. Discussion

The data presented here demonstrate that FCV was associated with at least some cases of FCGS and gingivitis, and that molnupiravir treatment of such cases was an effective cure of both virus shedding and clinical signs.

The fulfilment of Koch’s postulates for FCGS require the formal experimental inoculation of specific-pathogen-free laboratory (SPF) cats under controlled conditions. Some previous attempts to prove a causal effect of FCV failed to satisfy Koch’s postulates in that although some FCV strains taken from cats with FCGS caused acute gingivitis, they failed to consistently reproduce chronic stomatitis in experimental infections of SPF cats [30,31]. However, it has previously been reported that FCV strains from cats in FCGS outbreaks did cause what Reubel et al. [10] referred to as “acute faucitis” in experimentally-infected laboratory cats, although inflammation was still present as long as eight weeks post-infection in one cat. The findings of our study showed that FCV was significantly associated with FCGS. Not only did FCGS cats recover when FCV was eliminated, but three cats reinfected by their housemates relapsed.

However, our numbers were small and it must be borne in mind that there are many causes of feline oral inflammatory disease and that FCV is only one of them. It is vital that the diagnosis is correct; gingivitis secondary to dental disease will not respond to antiviral treatment. Although the teeth of most of the cats in our study appeared to be healthy, unfortunately the cats did not all have full-mouth dental radiography. Full-mouth dental radiography is particularly important because clinically normal-appearing teeth may have significant pathology, as was discovered in Cat 8 when she appeared to have recovered a second time. She was already being anaesthetised to be spayed so the opportunity presented itself for dental radiography, which revealed alveolar bone loss and a neck lesion. Cats with FCGS are significantly more likely than cats without FCGS to have alveolar bone loss [8]. Therefore, another criticism of our study could be that our assessment of clinical improvement was largely based upon gross, rather than radiographic, examination of the mouth.

An unexpected finding was that gingival hyperplasia resolved in ten cats following antiviral treatment; previously such lesions required surgical removal [59]. Hyperplasia took some weeks to resolve after the end of FCV shedding and occasionally left a faint discoloured tide mark on the teeth (Figure 3e). We are unable to give precise figures for how long it takes for oral mucosae to recover after the end of FCV shedding, but Cat 5’s gingival hyperplasia had fully resolved within eight weeks of beginning the antiviral, and the mouth of Cat 16 was considerably improved by five weeks, and completely healed within seven weeks of beginning treatment.

One limitation of the study is that evidence for recovery tended to be confined to oral inflammation scores and weight gain rather than a more formal assessment of how rapidly cats were experiencing relief from pain, using, for example, the Glasgow pain scale [60]. In retrospect, our inflammation scoring system should have included the presence or absence of hypersalivation as a pain indicator [5]. In a previous study, aggression was a prominent feature of cats with FCGS, and it resolved as the cat’s pain was reduced (Addie, personal observation) and we should have enquired about aggression. Future studies could involve giving the cat’s guardian a formal pain scoring sheet to indicate when concurrent pain medication can be reduced. Caregivers of cats treated with antivirals were reporting improvement in their cats’ appetite and demeanour within one to two weeks of starting treatment. As early as two weeks into treatment the guardian of Cat 16 reported that the cat could yawn without crying out in pain. Therefore, such a scoring system should be customised to the pain signs exhibited by the individual cat. Weight gain was a useful objective marker of pain reduction, assuming that a cat is likely to eat more when his/her mouth is less painful. In retrospect, weighing should have been done weekly.

Existing online dosing protocols seem to have been extrapolated from protocols optimised for treating FIP, but our data established that treating FCGS is fundamentally different in two key aspects: the cats require a larger dose, but for a much shorter duration determined by the cessation of FCV shedding. In this observational case series, the highest proportion of FCV clearance was observed among cats receiving a daily dose range of 40–55 mg/kg, which is approximately twice the daily recommended dose for treating FIP [52,53].

The duration of treatment required for calicivirus was shorter than that required to treat FIP, with most subclinically infected cats becoming FCV negative within two weeks, while cats with gingivitis required customised treatment until they tested FCV negative. Regarding treatment duration, there were two flaws in how the cases reported here were managed: first that initially cats were treated based upon the continued presence of gingival inflammation rather than the end of FCV shedding, and second, that cats were not FCV tested more frequently during treatment. Therefore, some cats were treated longer than necessary, although no adverse effects other than occasional vomiting were noted either in the cats or any kittens born to them.

Given that full mouth extractions cure 80% of FCGS cases [35], it may be wondered whether extracting teeth from three cats, rather than the antiviral, had affected delayed recovery. This was only possible in Cat 7, whose teeth were extracted 11 days prior to the antiviral, and is unlikely in Cats 15 and 16, whose extractions had occurred two months and over four years prior to healing.

Cats housed on their own or with FCV-negative housemate cats did not relapse, which rules out any question of the virus having gone latent or being below detectable levels. With the exception of three reinfected cats, sustained recovery was documented for up to 18 months.

The half-life of FCV shedding is 75 days [61], so it might be argued that FCV shedding ceased naturally and that the cats’ recovery was not due to the antiviral drug. This theory is highly improbable since FCGS cases tend to be persistently infected with calicivirus [31]. In FCGS cases the virus has found an antigenic niche unrecognisable to the immune system of the individual cat [31], making spontaneous clearance within the observation period unlikely. Furthermore, clinical signs resolved relatively rapidly following successful antiviral treatment, in contrast to the duration of suffering prior to antiviral treatment, which formed a retrospective control period for each individual cat, when they had not responded to conventional treatment. Clinical signs recurred upon three cats being reinfected by in-contact cats, suggesting that FCV was the cause of their clinical signs rather than an opportunistic infection. In addition, with one exception, FCV was eliminated before a clinical recovery occurred, not after recovery, as might be expected if virus infection was simply opportunistic.

The sensitivity of the FCV tests used could be questioned, i.e., could post-treatment negative results have been falsely negative? However, all except two cats were tested by virus isolation from the University of Glasgow Veterinary Diagnostic Services Laboratory [28], where, after several days of cell culture, if uncertainty remains as to whether a cytopathic effect is present then a sub-culture is performed; this ensures that false negative results are rare. Only two cats were tested by RT-qPCR. Unfortunately, IDEXX Laboratories do not routinely report cycle threshold (C*_T_*) values, but they recovered the C*_T_* value for Cat 15, which had a very low viral load. IDEXX Laboratories run controls for PCR inhibition but do not report the results of such controls unless asked. In addition to PCR inhibition with RT-PCR tests it is possible that primers and/or probe occasionally miss some field strains of FCV.

A major limitation of this study is that it was a real-life observational case series, conducted mostly in a working breeding cattery, and empirical in nature, where the priority was to cure cats suffering from FCGS, rather than a prospective, double-blinded and controlled study, which would have been the ideal. However, using an individual cat’s duration of suffering prior to treatment, as shown in Table 1, is a recognised valid retrospective control [62]. This approach reduces confounding factors such as breed, food and environment. Prior to molnupiravir most of the cats had suffered for months, and two cats suffered for over a year, whereas after antiviral treatment clinical improvement was sometimes reported as early as two weeks.

Whilst we acknowledge that the open cattery environment represents an inherent limitation of this research and led to reinfection and relapse of three cats before they could be neutered and rehomed, it also reflects the real-world conditions under which antiviral treatment of FCV-associated disease is likely to be applied in breeding or rescue cattery settings. Increased social contact with other cats and associated risk of viral re-exposure were unavoidable given the operational requirements of a working breeding cattery. As such, the findings retain clinical relevance and provide valuable insights into the challenges of sustained FCV management in an active working cattery.

A consequence of the empirical nature of the study was that cats were given varying antiviral doses for varying lengths of time, complicating interpretation. Cats were treated, tested for calicivirus, and the dose was increased or a longer treatment course started if the virus was still present. Gaps in molnupiravir treatment occurred in eight cats awaiting FCV test results. One curious observation was that Cats 33, 13 and 38 tested FCV negative in as few as 5, 5, and 6 days of treatment after gaps in treatment of 9, 19 and 7 days. In Cat 33, at the end of a 10-day treatment, FCV was viable and able to cause cytopathic effects in cell culture in only two days, yet following a 9-day treatment gap the cat ceased shedding virus after only five more days of molnupiravir. One possible explanation for these cats ceasing shedding the virus after shorter courses of treatment could be that the antiviral drug persisted in the salivary gland or gingiva during the treatment gap. The half-life of molnupiravir and its metabolite (N4-hydroxycytidine, NHC, EIDD-1931) in serum is around 12 hours [63,64]. Given the short half-life of molnupiravir, twice daily dosing would appear to be the ideal; however, dosing a cat with a painful mouth is not easy and compliance in administering the treatment outweighs the ideal. In our study, cattery cats were dosed once daily instead of twice daily. Twice-daily dosing possibly would have reduced the duration of treatment required; we did not examine that possibility. Circulating NHC is taken up into cells where it is phosphorylated to NHC-triphosphate (NHC-TP, EIDD-2061). The half-life of NHC-TP persists longer in peripheral blood mononuclear cells than in serum [63]. Another possibility is that the virus had been weakened by the previous course of treatment, which may have been the case in Cat 9. After 15 days of 59 mg/kg q24h molnupiravir, Cat 9 was still shedding virus, and there was a treatment gap of 11 days, then another 10 days of antiviral at the same dose. An oral swab taken on Day 34 finally grew virus after 13 days of culture and sub-culture, indicating a very low viral load or low viral pathogenicity, and the next swab on Day 58 was negative.

The third possible explanation is that the virus might have mutated and become visible to the immune system, enabling the cat to make antibodies able to neutralise the virus [64,65]. Whether the molnupiravir metabolite NHC-TP, which is mutagenic to viruses [66], hastened this process is unknown.

In the breeding cattery FCV outbreak described here, a surprisingly large percentage (37%: 14/38) of FCV-infected cats developed gingivitis and FCGS (as opposed to only upper respiratory signs). Any breed of cat can be affected by gingivitis, but those with the highest prevalence include Siamese [67,68], Maine Coon [37,67,68] and British Shorthairs [67]. Whether this higher breed prevalence is a reflection of genetic predisposition, or merely a consequence of the popularity of those breeds, is unclear. Seven of the 14 cattery cats with gingivitis were Maine Coons and the severity of their gingivitis seemed worse than in the British Shorthair/Longhair cats. However, our numbers were very small; so, this is an observation rather than a conclusion.

It is unknown why most FCV-infected cats appear to be asymptomatic while others develop gingivitis. This outbreak followed a change in vaccine from one containing inactivated calicivirus, to one containing a live F9 strain. In “original antigenic sin” theory when a vaccinated or previously-infected cat is reinfected with a new virus strain that has a slightly different epitope, the immune system relies on memory of the earlier infection or vaccine, rather than mounting another primary or secondary response to the new epitope, which would allow faster and stronger responses [69]. In this scenario, antibodies elicited against the original strain fail to neutralise the new virus strain. FCV strains have been shown to evolve within living cats; the virus mutates rapidly to outmanoeuvre the development of neutralising antibodies [65,70]. Poulet et al., 2000 [31] showed that strains of FCV taken from cats with FCGS were remarkably resistant to neutralisation by a panel of antibodies, and were particular to the individual cat from which they were isolated.

Vaccination failed to protect the cattery cats from either FCV infection or the development of gingivitis. Outbreaks of FCV have been documented to be due to vaccinal virus [71]; therefore, we now use only inactivated FCV vaccines in the cattery and in FCGS-recovered cats.

Another criticism of our study could be that very few blood tests were performed on molnupiravir-treated cats to check for adverse events due to the drug. In those cats that were blood tested, no haematological or biochemical abnormalities were noted. Few adverse events have been reported during longer 12-week treatments for feline infectious peritonitis [51,52,53,72]. NHC-TP is mutagenic not only to viruses but also to mammalian cells [66], but no neoplasia adverse effects occurred in over 500 feline infectious peritonitis cases treated with molnupiravir (Dr Okihiro Sase, personal communication).

NTZ requires further investigation as a possible alternative to molnupiravir, or as a possible synergistic antiviral drug. NTZ alone failed to stop FCV shedding in three cats with doses of 31–48 mg/kg q24h. NTZ adverse events were noted in two cats. Cat 7 vomited, so treatment could not continue, and Cat 8 developed diarrhoea, which was partially managed by treating her every 48 h, but which prevented a third course being given.

Towards the end of this study EIDD-1931 became available, and a dose of 32 mg/kg q24h stopped the shedding of FCV within 11 days in the single subclinically infected cat. Cats must metabolise molnupiravir into EIDD-1931, and the rate of metabolising molnupiravir varies between individual cats [64], so using EIDD-1931 directly is logical. However, EIDD-1931 is less bioavailable orally than molnupiravir [73]. Given the different bioavailability of EIDD-1931 and molnupiravir it is likely that the optimal dose and duration to eliminate FCV will need to be established *in vivo,* and it may differ from the protocol established here. Nevertheless, now that the approximate dosages for successful elimination of the virus are established, hopefully this study will prevent unnecessary continued molnupiravir treatment long after it is apparent that it is not effective against the virus infecting an individual cat, as occurred with Cat 8.

## 5. Conclusions

This study provided evidence that cats with gingivitis can recover after treatment to stop calicivirus shedding, thus avoiding the painful removal of healthy teeth. The most effective dose of molnupiravir was 40–55 mg/kg; too low a dose failed to reliably stop calicivirus shedding.

Subclinically infected cats cease shedding calicivirus within 14 days. Cats with chronic inflammatory conditions require tailored individual treatment, ceasing the antiviral treatment when the cat tests FCV negative. If the cat does not test FCV negative within 40 days of treatment, another antiviral such as NTZ or feline interferon omega [33,46,47,48,49] may need to be considered.

FCV shedding ceased before clinical signs of inflammation resolved; therefore, anti-inflammatory treatment may be required post-antiviral. Pain medication should be carefully calibrated for each individual cat during the course of their recovery.

FCV vaccination prevented neither infection nor gingivitis; therefore, in retrospect, we should have tested all FCV-infected cats in the cattery simultaneously and quarantined infected cats to prevent reinfection and relapse of cured cats.

Due to the different pharmacokinetics of molnupiravir and EIDD-1931 future studies will be required to establish more precise treatment protocols than those presented here.

## Figures and Tables

**Figure 4:**
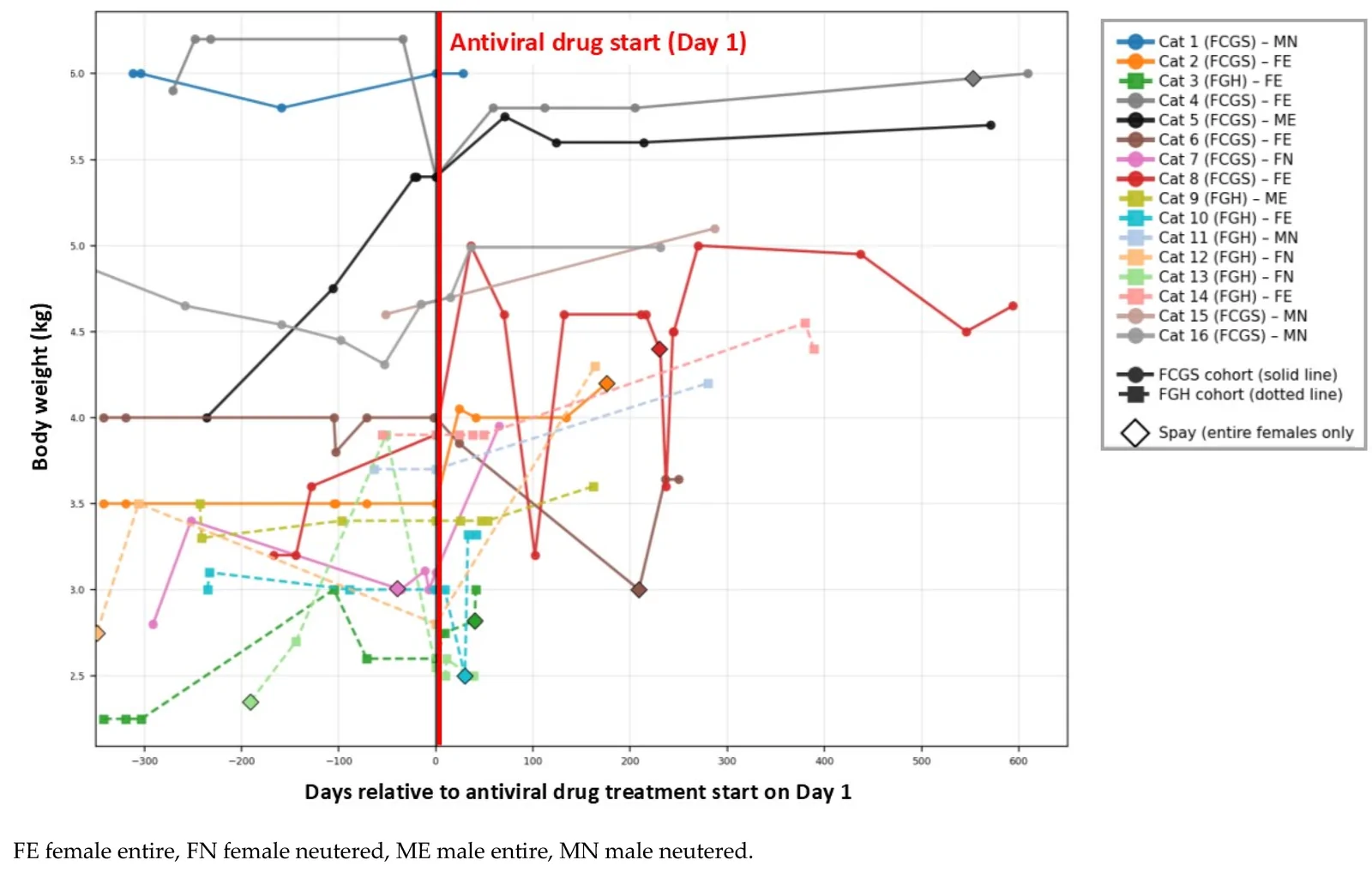
Cat weights before and after antiviral treatment cleared feline calicivirus. The aim of this graph is to show weight gain after cats cleared FCV infection. The weights of the gingivitis cats were plotted (on the y axis) against the days relative to the beginning of the antiviral that effectively stopped FCV shedding, i.e., molnupiravir treatment (Day 1 indicated by the vertical red line) in all cats except Cat 8, whose virus only responded to nitazoxanide, so Day 1 represents the start of the first course of nitazoxanide in her case. This graph shows that eleven cats gained weight when they stopped shedding feline calicivirus and their gingival inflammation resolved. Cats 5, 6 and 8 were reinfected and relapsed, Cat 8 gained weight after two NTZ treatments, Cat 6 did not gain weight after either treatment, and Cat 5 gained weight after his first treatment, but no weight was available after his second.

## Data Availability

The data are presented within this manuscript.

## Supplementary Materials

The following supporting information can be downloaded at: https://www.mdpi.com/article/10.3390/ani16203265/s1. Table S1. Timeline of cattery feline calicivirus outbreak. Table S2. Details of cats with gingivitis and vaccine history prior to molnupiravir treatment.

## Abbreviations

The following abbreviations are used in this manuscript:

AOG	Adult-onset gingivitis
BLH	British Longhair cat
BSH	British Shorthair cat
CI	Confidence interval
C*_T_*	Cycle threshold
DSH	Domestic shorthair cat
EIDD-1931	Active metabolite of molnupiravir (NHC)
EIDD-2801	Molnupiravir
FCGS	Feline chronic gingivostomatitis
FCoV	Feline coronavirus
FCV	Feline calicivirus
FGH	Feline gingival hyperplasia
FIP	Feline infectious peritonitis
FME	Full mouth extraction
GA	General anaesthetic
h	Hours
IQR	Interquartile range
MCO	Maine Coon cat
mg/kg	Milligrams per kilogram
n	Number
NHC	β -D-N4-hydroxycytidine
NHC-TP	β-D-N4-hydroxycytidine triphosphate
NTZ	Nitazoxanide
q12h	Every 12 hours
q24h	Every 24 hours
RT-qPCR	Reverse transcriptase quantitative polymerase chain reaction
SPF	Specific pathogen free
URT	Upper respiratory tract
