# Supplementary material for: The Most Effective Molnupiravir Dosage for Eliminating Feline Calicivirus from Cats With and Without Chronic Gingivostomatitis
Source: Animals (Basel). 2026 Oct 16;16(20):3265. doi: 10.3390/ani16203265 (PMC13700706; doi:10.3390/ani16203265)
Supplement: Supplementary file 1 [file animals-16-03265-s001.zip › animals-4519842-supplementary.pdf]

Supplementary Table S1. Timeline of cattery feline calicivirus outbreak

Year	Month	Event
2023	April	The index gingivitis case in the cattery presented with Grade 1 gingival inflammation two days after a live calicivirus F9 strain booster vaccine (Nobivac Tricat Trio™ MSD, London, UK). However, his data were excluded because he was not tested for feline calicivirus (FCV).
	May	The second cattery case was diagnosed with feline chronic gingivostomatitis (FCGS), 264 days after his previous vaccine (Cat 1, Table 1). When he didn't respond to treatment with mefloquine and non-steroidal anti-inflammatory drugs a dental examination under general anaesthetic was performed; his teeth were found to be perfectly healthy so they were not extracted (Table 1). Histopathology of gingival biopsies was reported as lymphoplasmacytic gingivitis.
2024	June	Cats 5 & 8 presented with FCGS.
	July	Cat 2 presented with FCGS.<br>Throughout the summer of 2024 many kittens experienced severe upper respiratory tract (URT) signs to the extent that the cattery owner set up an oxygen tent for the most severely affected.
	August	Cat 8 began her first nitazoxanide (NTZ) treatment for FCGS.
	September	Encouraged by the recovery of Cat 8 following NTZ treatment in September 2024 (Figure 2 (c)(d)), NTZ was subsequently administered to four other cats (see Table 1), but discontinued in one due to a vomiting adverse effect.<br>Vaccines containing live FCV strain F9 administered to 24 kittens (six different litters); 14 suffered adverse reactions, 11 had severe URT signs; the first kitten died and necropsy revealed severe pneumonia.
	October	Three more kittens died.<br>Cat 3 presented with FGH, Cats 4, 6, and 7 with FCGS. However, Cat 7 vomited the NTZ pills, Cats 2, 3, and 6 remained FCV positive and symptomatic (Table 1), therefore, NTZ was discontinued. Molnupiravir was then used.
	November	A mixed infection of two plaque sizes of FCV was isolated in cell culture [54] from the lungs of two faded kittens which had been post-mortemed; small and minute plaques similar to the plaque sizes of strains from the F9 vaccine vial strain and larger plaques possibly from a field infection (larger plaque sizes indicate more virulent calicivirus [55]).<br>Cat 8 was reinfected and relapsed with FCGS.
2025	January & February	Return to vaccine containing inactivated calicivirus antigens 431 and G1 strains (Purevax RCP™, Boehringer Ingelheim, Lyon, France).<br>Widespread FCV testing of cattery cats was implemented and continued henceforth.
	March	From March 2025 three cats with severe FCV-related URT signs were treated with molnupiravir.
	April	Cat 14 presented with FGH.
	May	Cat 9 presented with feline gingival hyperplasia (FGH).
	June	Cat 10 presented with FGH. Cat 5 reinfected and relapsed with FGH.<br>Data analysis established that the optimal dose of molnupiravir is 40–55 mg/kg q24h.
	August	Cats 11, 12, and 13 presented with FGH.
	September	Cat 6 relapsed with adult-onset gingivitis.
	December	Second relapse of Cat 8.
2026	January - June	The colony continues to have regular veterinary dental checks amongst all the cats. Cats are checked daily and weighed monthly by the breeder. The attending veterinary surgeons approved a protocol to prescribe EIDD-1931 to all queens demonstrating clinical signs of gingivitis or URT contingent upon a confirmed FCV-positive laboratory test. Kitten morbidity and mortality have been dramatically reduced as a consequence of this approach.

This table shows the timeline of the cattery FCV and gingivitis and upper respiratory tract infection outbreak in the Maine Coon and British Shorthair breeding cattery.

Supplementary Table S2. Details of cats with gingivitis and vaccine history prior to molnupiravir treatment.

Cat	Breed	Sex	Age (years) when gingivitis diagnosed	FCV vaccine(s) prior to onset of gingivitis	Interval from vaccine to gingivitis diagnosis (days)
1	MCO	MN	1.0	Nobivac Tricat Trio.	264
2	MCO	FE	1.2	Nobivac Tricat Trio.	10
3	BLH	FE	1.0	Nobivac Tricat Trio.	11
4	MCO	FE	4.5	Nobivac Tricat Trio, previously Versifel CVR.	215
5	MCO	ME	0.6	Purevax RCP.	127
5-r*			1.6	Purevax RCP.	475 (relapse)
6	BSH	FE	2.4	Purevax RCP. Tricat Trio in previous year.	345
6-r			3.3	Purevax RCP booster given after the first episode.	265 (relapse)
7	BSH	FE	2.4	Nobivac Tricat Trio.	73
8	MCO	FE – FN**	0.8	The cat's 2nd kitten vaccine was Purevax RCP, her 1st dose was Nobivac Tricat Trio.	187 (1st FCGS)
8-r			1.1	No booster vaccine was administered; only the kitten course shown above.	324 (relapse)
9	BSH	ME	3.2	Nobivac Tricat Trio.	293
10	BSH	FE	3.1	Nobivac Tricat Trio.	311
11	BSH	MN	3.6	Leucofeligen.	363
12	MCO cross	FN	1.9	Nobivac Tricat Trio.	279
13	BSH	FE	1.1	1st dose: Nobivac Tricat Trio. 2nd dose: Purevax RCP.	324
14	MCO	FE	0.8	Purevax RCP.	193
15	DSH	MN	1.9	Unknown (possibly done in the shelter): FCGS found on day of rabies vaccine in 2024.	Unknown
16	DSH	MN	4.6	FVRCP unknown brand.	3 years

* an -r after a cat's reference number indicates re-infection and relapse

** Cat 8 was spayed during her second relapse.

FE: female entire

FN: female neuter

ME: male entire

MN: male neuter

BLH: British longhair

BSH: British shorthair

DSH: Domestic shorthair

MCO: Maine Coon

This table shows the breeds of the cats with gingivitis; their sex, ages when gingivitis was first noticed; their vaccination status; and how many days had elapsed between their most recent vaccination prior to the gingivitis diagnosis. Cat 11 was diagnosed on the day he was presented for his annual booster vaccine.
